# Deformable Pyramid Sparse Transformer for Semi-Supervised Driver Distraction Detection

**DOI:** 10.3390/s26030803

**Published:** 2026-01-25

**Authors:** Qiang Zhao, Zhichao Yu, Jiahui Yu, Simon James Fong, Yuchu Lin, Rui Wang, Weiwei Lin

**Affiliations:** 1 School of Communication and Information Engineering, Shanghai University, Shanghai 200444, China; 20820142@shu.edu.cn (Q.Z.); rwang@shu.edu.cn (R.W.); 2 Department of Computer and Information Science, Faculty of Science and Technology, University of Macau, Macau, China; yc57985@um.edu.mo (Z.Y.); mc35112@um.edu.mo (J.Y.); 3 School of Computer Science and Engineering, Macau University of Science and Technology, Macau, China; 3220001931@student.must.edu.mo; 4 School of Computer Science and Engineering, South China University of Technology, Guangzhou 510006, China; linww@scut.edu.cn

**Keywords:** driver distraction detection, semi-supervised learning, teacher–student framework, deformable feature fusion, multi-scale feature alignment, object detection, YOLO-based detection, intelligent transportation systems, driver monitoring systems

## Abstract

Ensuring sustained driver attention is critical for intelligent transportation safety systems; however, the performance of data-driven driver distraction detection models is often limited by the high cost of large-scale manual annotation. To address this challenge, this paper proposes an adaptive semi-supervised driver distraction detection framework based on teacher–student learning and deformable pyramid feature fusion. The framework leverages a limited amount of labeled data together with abundant unlabeled samples to achieve robust and scalable distraction detection. An adaptive pseudo-label optimization strategy is introduced, incorporating category-aware pseudo-label thresholding, delayed pseudo-label scheduling, and a confidence-weighted pseudo-label loss to dynamically balance pseudo-label quality and training stability. To enhance fine-grained perception of subtle driver behaviors, a Deformable Pyramid Sparse Transformer (DPST) module is integrated into a lightweight YOLOv11 detector, enabling precise multi-scale feature alignment and efficient cross-scale semantic fusion. Furthermore, a teacher-guided feature consistency distillation mechanism is employed to promote semantic alignment between teacher and student models at the feature level, mitigating the adverse effects of noisy pseudo-labels. Extensive experiments conducted on the Roboflow Distracted Driving Dataset demonstrate that the proposed method outperforms representative fully supervised baselines in terms of mAP@0.5 and mAP@0.5:0.95 while maintaining a balanced trade-off between precision and recall. These results indicate that the proposed framework provides an effective and practical solution for real-world driver monitoring systems under limited annotation conditions.

## 1. Introduction

Driver distraction has become one of the leading causes of traffic accidents worldwide, posing a serious threat to road safety and public health. With the rapid development of intelligent transportation systems and advanced driver assistance systems (ADASs), reliable driver monitoring has emerged as a key component for improving driving safety and reducing accident risks. Among various driver monitoring tasks, driver distraction detection plays a critical role by identifying unsafe behaviors such as using a mobile phone, drowsiness, head-down operations, or inattentive gaze patterns, enabling timely warnings or intervention mechanisms.

Early driver distraction detection approaches primarily relied on handcrafted features combined with traditional machine learning classifiers, such as support vector machines and random forests. These methods typically exploited visual cues related to head pose, eye closure, or hand position but suffered from limited robustness under complex lighting conditions, occlusions, and large inter-driver variability. With the advancement of deep learning, convolutional neural networks (CNNs) and transformer-based models have significantly improved the performance of visual perception tasks. In particular, object detection frameworks based on the YOLO family have demonstrated strong real-time performance and competitive accuracy in driver behavior analysis tasks [1,2,3]. Recent detector variants further enhance spatial attention and feature aggregation, enabling more reliable detection of fine-grained driver actions [4,5,6].

Despite these advances, most existing driver distraction detection methods rely heavily on fully supervised learning paradigms, which require large-scale, accurately annotated datasets. In real-world driving scenarios, collecting and labeling such datasets is both time-consuming and expensive, especially for rare or ambiguous distraction behaviors. Moreover, annotation inconsistency and class imbalance further limit the scalability and generalization capability of fully supervised models. As a result, detection models trained on limited labeled data often exhibit performance degradation when deployed in complex or unseen environments.

Semi-supervised learning has recently gained increasing attention as an effective solution to alleviate annotation dependency by leveraging abundant unlabeled data together with a small labeled subset. In particular, teacher–student learning frameworks have been widely adopted in semi-supervised object detection, where a teacher model generates pseudo-labels to guide the training of a student model. However, pseudo-label quality remains a major challenge, as early-stage teacher predictions are often noisy and uneven across categories. Without appropriate control mechanisms, erroneous pseudo-labels can propagate through training, leading to model drift and unstable convergence. Several studies have attempted to address this issue through confidence thresholding or consistency regularization, but most methods still rely on fixed global thresholds and lack adaptive strategies tailored to category characteristics and training dynamics.

In parallel, accurately detecting driver distraction requires strong sensitivity to subtle and localized visual cues, such as eye closure, gaze direction, and small hand movements. Conventional convolutional feature pyramids may struggle to align semantic information across scales, particularly under occlusion and varying illumination. Transformer-based feature fusion techniques have shown promise in enhancing global context modeling, yet their computational cost and dense attention mechanisms limit their applicability in real-time driving scenarios. Sparse attention and deformable alignment mechanisms provide a potential solution by selectively focusing on informative regions while maintaining computational efficiency.

Motivated by the above challenges, this work addresses two key limitations in existing driver distraction detection systems: (1) the heavy reliance on large-scale labeled data, and (2) the insufficient representation of fine-grained, multi-scale driver behaviors under complex driving conditions. To this end, we propose an adaptive semi-supervised driver distraction detection framework that integrates teacher–student learning with deformable pyramid feature fusion. The proposed approach introduces adaptive pseudo-label optimization strategies to dynamically control pseudo-label quality and training stability, while a Deformable Pyramid Sparse Transformer enhances cross-scale feature alignment and local semantic sensitivity. By jointly addressing learning efficiency and feature representation robustness, this research provides a scalable and practical solution for real-world driver monitoring systems, particularly in scenarios where annotation resources are limited.

## 2. Related Work

### 2.1. Semi-Supervised Object Detection

Semi-supervised object detection (SSOD) has made significant progress by incorporating unlabeled data into training to reduce reliance on costly annotations. The teacher–student paradigm remains one of the most widely adopted frameworks in SSOD. Early success was demonstrated by methods that improved pseudo-label quality and stability. For instance, CSD (Consistency-based Semi-supervised Detection) enforces consistency between predictions for weak and strong augmentations, which enhances robustness in pseudo-labeling [7]. STAC (Self-Training with Augmentation Consistency) further demonstrated the value of strong data augmentation and confidence thresholding for pseudo-label selection in object detection tasks [8]. More recently, Unbiased Teacher introduced momentum updates and confidence threshold scheduling to mitigate pseudo-label bias, substantially improving state-of-the-art SSOD results on standard benchmarks such as MS-COCO [9]. Building on this line, SoftTeacher proposed a soft instance assignment to exploit pseudo-labels with lower confidence scores without being overly restrictive [10].

Most teacher–student models use fixed global confidence thresholds for pseudo-label selection, which can be suboptimal due to class imbalance and dynamic training behavior. To address this issue, adaptive thresholding mechanisms have been explored. For example, Adaptive Thresholding SSOD dynamically adjusts thresholds based on category statistics and prediction entropy to improve pseudo-label precision and recall [11]. Despite these advances, most approaches still struggle when pseudo-label noise is high, particularly in fine-grained detection tasks such as behavior recognition

### 2.2. Adaptive and Robust SSOD Techniques

To enhance robustness in semi-supervised learning, several adaptive strategies have been developed. Meta-Regulated SSOD uses meta-learning to optimize pseudo-label selection criteria, leading to more reliable learning from unlabeled data [12]. LabelMatch addresses pseudo-label noise by integrating distribution alignment and loss re-weighting, achieving strong performance on both balanced and long-tail distributions [13]. In addition, adaptive augmentations such as region mixup and object-aware transformations have shown performance gains by regularizing the student model training [14,15]. From a robustness perspective, Noisy Student Training leverages large unlabeled sets with iterative self-training cycles to improve generalization [16].

Although these adaptive strategies improve SSOD generalization, most are evaluated on generic object detection benchmarks with relatively homogeneous classes and limited domain variation. When applied to complex, real-world detection scenarios, such as driver behavior analysis, the effectiveness of these techniques diminishes due to subtle object features and intra-class variance.

### 2.3. Transformer- and Attention-Based Feature Learning

Improving feature representation has been a key focus of recent object detection research. Transformer-based architectures have been widely adopted due to their ability to model global context. Deformable DETR introduced deformable attention modules to reduce computational cost while preserving performance gains from transformer mechanisms [17]. Beyond global attention, hierarchical transformers like Swin Transformer demonstrated strong multi-scale representation capability for dense prediction tasks [18]. To specifically support multi-scale fusion, Pyramid Vision Transformer (PVT) and its variants improved fine-grained localization by learning scale-aware features [19].

In semi-supervised contexts, integrating transformer representations within teacher–student frameworks has shown promise. For example, studies combining multi-scale transformer encoders with robust pseudo-labeling reported improved detection and segmentation performance in low-annotation settings [20,21]. These works underscore the potential of attention-oriented feature learning to complement semi-supervised strategies, especially in tasks demanding high sensitivity to localized cues.

### 2.4. Driver Distraction Detection

Driver distraction detection (DDD) plays a central role in modern driver monitoring systems and intelligent transportation applications, aiming to identify behaviors that may compromise driving safety, such as mobile phone usage, drinking, reaching behind, or fatigue-related actions. Due to its direct impact on road safety, DDD has attracted sustained research attention across computer vision, human behavior analysis, and intelligent vehicle communities.

Early studies on driver distraction primarily relied on hand-crafted visual cues and domain-specific heuristics. Typical approaches extracted features related to head pose, eye closure, gaze direction, and hand position, which were subsequently classified using traditional machine learning models [21,22]. Liang et al. [23] developed a method for real-time detection of cognitive distraction using support vector machines (SVM) and driver eye movement and driving performance data. The results showed that the SVM model could detect driver distraction with an average accuracy of 81.1%, which is better than the traditional logistic regression model. Although these methods are computationally efficient and interpretable, their performance is highly sensitive to illumination changes, occlusion, camera placement, and individual driver characteristics, limiting their robustness in real-world driving environments.

With the rapid development of deep learning, convolutional neural networks (CNNs) became the dominant paradigm for DDD. CNN-based classifiers and detection frameworks demonstrated substantial improvements by learning discriminative representations directly from raw images or video frames [24,25]. Li et al. [26] proposes a lightweight convolutional neural network, OLCMNet, incorporating an octave-like convolution mixed block, to detect driver distraction with limited computational resources and achieve a balance between accuracy and real-time performance on both self-built datasets and publicly available datasets. Chen et al. [27] proposed a fine-grained driver distraction detection method based on neural networks, fusing multi-source perceptual information related to the driver. It utilizes convolutional neural networks to extract high-dimensional mapping features for driver distraction detection. On a large-scale multimodal driver distraction dataset, the model achieves an average detection accuracy of 99.7796%. Meanwhile, the adoption of real-time object detectors, particularly YOLO-family models, enabled practical deployment by providing a favorable balance between accuracy and inference efficiency [28,29]. Rampavan et al. [2] proposed a YOLO detection algorithm based on an improved genetic algorithm (GA) and employs a network analysis (NAS) search strategy. Experimental results show that the proposed method has a smaller model size and better performance compared to existing single-stage and two-stage object detection models, demonstrating its effectiveness in designing driver distraction detection systems.

As the Transformer continues to gain popularity, more and more people are beginning to use Transformer structures and attention mechanisms for driver distraction detection [30,31]. Doshi [32] proposed a novel vision transformer architecture, Anchor-ViT, which combines learnable spatial anchors with a soft radial attention (SRA) mechanism to adaptively focus on key areas of the driver. On the State Farm and 100-Driver Distracted Driving datasets, Anchor-ViT achieves 5.2% higher accuracy than the baseline ViT model, effectively balancing the need for local sensitivity and comprehensive scene understanding. Qiao et al. [33] proposed a Driver Cognitive Distraction Detection (DCDD) model using driver gaze points captured by eye trackers and dashcam (DCI) images as input. They designed a generator eye-tracking heatmap, a fusion adversarial network (FAN), and a multi-view spatial channel network (MSCN) to help the Transformer model capture DCI and map features more comprehensively over time. Experiments show that the model achieves an accuracy of 96.42% while controlling model parameters and floating-point operations.

Despite the advancements made by convolutional neural networks and transformer-based models in driver distraction detection, practical distracted driving data design (DDD) remains challenging due to several fundamental issues often overlooked in general object detection benchmarks. First, the scarcity and cost of labeled data are major bottlenecks. Fine-grained distracted driving behavior requires precise temporal and spatial annotations, which are both expensive and time-consuming to acquire on a large scale. Consequently, many datasets have limited labeled samples and biased coverage of driver populations. Fully supervised models trained under such conditions are prone to overfitting and often fail to generalize to unseen drivers or environments. Second, the inherent intra-class variability and long-tail distribution of driver behavior data are inherent characteristics. Due to differences in body size, posture, driving habits, and interaction patterns, the same distracted driving behavior may exhibit different characteristics across different drivers. Furthermore, safety-critical behaviors typically occur less frequently than normal driving, leading to severe class imbalance. These characteristics pose challenges to both classification and detection models, as the dominant class often masks the behavior of the minority class during training, resulting in unstable decision boundaries and reduced recall for rare events. Third, subtle and localized visual cues under complex conditions pose significant challenges to reliable detection. Many distraction behaviors are characterized by small or partially occluded areas in the image, such as hand-object interactions or changes in eye state. These cues vary in spatial location and scale depending on camera setup and driver posture. Traditional feature pyramids lack explicit mechanisms for establishing accurate cross-scale correspondences, while dense or global attention mechanisms introduce high computational costs, limiting their application in real-time vehicle systems.

To alleviate annotation limitations, semi-supervised and weakly supervised learning strategies have begun to emerge in driver distraction detection (DDD) research. These methods aim to improve the generalization ability of models with limited annotation budgets by utilizing large amounts of unlabeled in-vehicle driving data. Mohammed et al. [34] proposed a distracted driving behavior detection method based on a lightweight visual Transformer and utilize pseudo-label-based semi-supervised learning to train the model. The effectiveness of the proposed method is validated through comparison and evaluation with alternative fully supervised and semi-supervised methods. Chen et al. [35] proposed DSDFormer, which combines the advantages of Transformer and Mamba architectures through a dual-state domain attention (DSDA) mechanism and introduces an unsupervised temporal reasoning confidence learning (TRCL) method to optimize noise labels by leveraging the spatiotemporal correlations in video sequences. Extensive experimental results demonstrate that DSDFormer significantly improves the accuracy and robustness of driver distraction detection. However, many current semi-supervised DDD methods directly inherit pseudo-label selection strategies from general SSOD settings, relying on fixed or global confidence thresholds. This design fails to take into account the difficulty of category-related detection, individual differences among drivers, and long-tail distribution, which usually leads to high false tag noise and unstable trajectories.

Given these limitations, we propose a unified framework aimed at simultaneously improving the learning stability and feature representation capabilities of semi-supervised driver distraction detection. For learning, we introduce an adaptive semi-supervised strategy to address issues such as class-related detection difficulty, class imbalance, and pseudo-label noise. For representation, we design a Deformable Pyramid Sparse Transformer (DPST) to enhance cross-scale alignment and focus on semantically relevant local regions, thereby enabling more accurate modeling of fine-grained driver behavior under various conditions.

## 3. Methodology

### 3.1. Overall Framework of the Proposed Semi-Supervised Detection System

**Figure 1:**
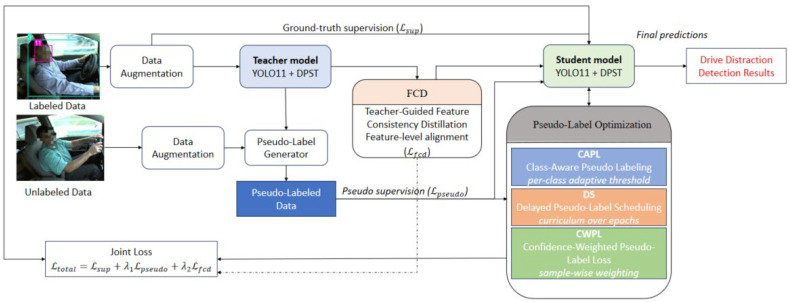
The Overall framework of our Semi-Supervised Detection System.

The proposed methodology adopts a semi-supervised object detection framework designed to effectively exploit a large volume of unlabeled driving behavior images together with a limited set of annotated samples, thereby enhancing the detection accuracy and robustness of driver distraction recognition. The overall architecture of the proposed system is illustrated in Figure 1. The training process follows a teacher–student paradigm and is divided into two sequential stages: teacher model guidance and student model self-learning.

In the first stage, the teacher model is trained using the available labeled data under a fully supervised setting to obtain stable detection capability. Once sufficiently converged, the teacher model is used to generate pseudo-labels for unlabeled driving behavior images. In the second stage, the student model is trained on both labeled samples and pseudo-labeled data, where the pseudo-supervision provided by the teacher is progressively incorporated. During this stage, several adaptive learning strategies—including dynamic loss weighting, delayed pseudo-label scheduling, and feature consistency constraints—are employed to improve training stability and mitigate the negative impact of noisy pseudo-labels.

**Figure 2:**
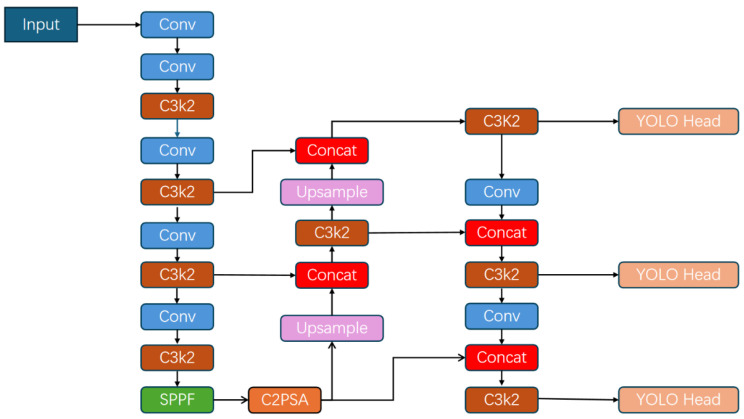
The structure of basic YOLOv11. C3K2 is the efficient convolution module proposed in YOLOv11. C2PSA is the cross-stage local spatial attention module proposed in YOLOv11, which enhances the spatial attention in the feature map and improves the model’s focus on important parts of the image.

For the detection backbone, we adopt the lightweight object detection architecture YOLOv11, which achieves a favorable trade-off between detection accuracy and computational efficiency through the introduction of efficient convolutional and attention-based modules [36]. Specifically, YOLOv11 incorporates the C3K2 module for efficient feature extraction and the C2PSA module for cross-stage local spatial attention, enabling enhanced focus on salient regions in the feature maps. The overall architecture of the baseline YOLOv11 detector is illustrated in Figure 2. To further strengthen the model’s ability to capture subtle driver behaviors, such as fine hand movements and gaze variations, a Deformable Pyramid Sparse Transformer (DPST) module is embedded into the detection network.

To suppress the accumulation and propagation of pseudo-label errors, particularly during the early stages of semi-supervised training, the proposed framework integrates three collaborative pseudo-label optimization mechanisms: Class-Aware Pseudo-Labeling (CAPL), Delayed Pseudo-Label Scheduling (DS), and Confidence-Weighted Pseudo-Label Loss (CWPL). These mechanisms jointly optimize pseudo-label selection, regulate the temporal introduction of pseudo-supervision, and dynamically reweight loss contributions based on pseudo-label confidence. Furthermore, to strengthen the guidance provided by the teacher model, a lightweight Feature Consistency Distillation (FCD) module is introduced. This module enforces semantic consistency between intermediate feature representations of the teacher and student networks, facilitating effective knowledge transfer and improving representation stability.

As illustrated in Figure 1, all components are integrated into an end-to-end trainable semi-supervised learning pipeline. This unified design enables the student model to converge robustly even under uneven pseudo-label quality and feature-level noise, which are common challenges in real-world driving scenarios.

It is worth noting that, unlike conventional semi-supervised detection methods that rely on a global fixed confidence threshold, the proposed framework employs category-specific adaptive thresholds during pseudo-label generation and screening. This design allows the model to account for inter-class variability in detection difficulty—for example, applying different confidence criteria to categories such as making a phone call and looking forward—thereby alleviating overfitting caused by class imbalance. In addition, a phased pseudo-label scheduling strategy is introduced to synchronize the proportion of unlabeled samples with the prediction stability of the teacher model. By dynamically balancing pseudo-label quantity and quality throughout training, the proposed framework effectively improves driver distraction detection performance while substantially reducing the need for large-scale manual annotation.

### 3.2. Pseudo-Label Optimization Strategies (PLOS)

In semi-supervised object detection, the effectiveness of learning from unlabeled data is highly dependent on the quality of pseudo-labels, the timing of their incorporation, and the weighting strategy used during optimization. Noisy or prematurely introduced pseudo-labels can significantly degrade training stability and lead to suboptimal convergence. To address these challenges, we design a unified Pseudo-Label Optimization Strategy (PLOS) composed of three complementary components: Class-Aware Pseudo-Labeling (CAPL), Delayed Pseudo-Label Scheduling (DS), and Confidence-Weighted Pseudo-Label Loss (CWPL). Together, these mechanisms regulate pseudo-label selection, temporal participation, and loss contribution, forming an adaptive pseudo-supervised learning scheme that enhances robustness against pseudo-label noise.

#### 3.2.1. Class-Aware Pseudo-Labeling (CAPL)

In conventional semi-supervised detection frameworks, a global confidence threshold *τ* is typically applied to filter teacher predictions. However, in driver distraction detection, different behavior categories exhibit substantial variation in visual separability. For instance, safe driving samples often present stable global patterns, whereas behaviors such as holding a phone or head-down operation rely on subtle and localized cues. Applying a unified threshold therefore leads to an imbalanced pseudo-label distribution across classes and premature elimination of hard yet informative samples.

To alleviate this issue, we propose Class-Aware Pseudo-Labeling (CAPL), which dynamically assigns confidence thresholds on a per-class basis. Let *c* ∈ {1, …, *C*} denote a behavior category. For each class, we compute the statistical confidence distribution of teacher predictions on the labeled set and define the adaptive threshold as

$$\tau_{c}=\mu_{c}+\alpha \cdot \sigma_{c}$$

where $\mu_{c}$ and $\sigma_{c}$ represent the mean and standard deviation of confidence scores for class *c*, respectively, and *α* is a tunable hyperparameter controlling strictness. Classes with stable predictions naturally receive higher thresholds, while those with larger variance are assigned lower thresholds to preserve potentially correct samples.

Given the teacher-generated predictions $\left\{\left(x_{i},{\hat{y}}_{i},p_{i}^{c}\right)\right\}$, the retained pseudo-label set for class *c* is defined as

$$P_{c}=\left\{\left(x_{i},{\hat{y}}_{i}\right)|p_{i}^{c}\geq \tau_{c}\right\}$$

These thresholds are updated periodically during training, enabling continuous adaptation to evolving teacher confidence. CAPL effectively mitigates class imbalance and improves the diversity and reliability of pseudo-supervision.

#### 3.2.2. Delayed Pseudo-Label Scheduling (DS)

At early training stages, the teacher model is insufficiently optimized, resulting in pseudo-labels with high uncertainty. Directly incorporating such pseudo-labels may bias the student toward erroneous feature distributions. To prevent this, we introduce a Delayed Pseudo-Label Scheduling (DS) strategy that progressively increases the influence of pseudo-labeled data as training proceeds.

We define a time-dependent weighting factor:

$$\lambda_{t}=min\left(1,\frac{t}{T_{d}}\right),$$

where *t* is the current training epoch and $T_{d}$ is a delay threshold. When *t* < $T_{d}$, training relies solely on labeled data. As *t* increases beyond $T_{d}$, pseudo-labels gradually participate until full contribution is achieved. This slow-start mechanism effectively suppresses early noise accumulation.

In parallel, the teacher model parameters are updated using an exponential moving average (EMA) of the student parameters:

$$\theta_{t}^{teacher}=\beta \theta_{t-1}^{teacher}+\left(1-\beta \right)\theta_{t}^{student}$$

where *β* controls the update smoothness. The combination of EMA and DS ensures that pseudo-labels are generated by a stable teacher while being introduced at appropriate training stages, yielding robust optimization dynamics.

#### 3.2.3. Confidence-Weighted Pseudo-Label Loss (CWPL)

Although CAPL and DS improve pseudo-label quality at the dataset and temporal levels, individual pseudo-labels still exhibit varying reliability. Treating all pseudo-labels equally may overemphasize noisy samples and dilute high-confidence supervision. To address this issue, we introduce a Confidence-Weighted Pseudo-Label Loss (CWPL) that assigns sample-level weights based on confidence.

For a pseudo-labeled sample $\left(x_{i},{\hat{y}}_{i},p_{i}^{c}\right)$, where $c_{i}\in [0,1]$ denotes confidence, the weighting factor is defined as

$$w_{i}=\gamma \cdot c_{i}+\left(1-\gamma \right)$$

where γ∈[0,1] controls the balance between confidence emphasis and baseline contribution. The pseudo-label loss is then formulated as

$$L_{pseudo}=\frac{1}{N}\sum_{i=1}^{N}w_{i}\cdot l\left(f\left(x_{i}\right),{\hat{y}}_{i}\right)$$

where *ℓ*(⋅) denotes the standard YOLO detection loss composed of bounding-box regression, distribution focal loss, and classification terms. CWPL sharpens gradients toward high-confidence pseudo-labels while naturally suppressing unreliable ones, without altering the underlying optimization structure of YOLO.

### 3.3. Teacher-Guided Feature Consistency Distillation (FCD)

Beyond output-level supervision, intermediate feature representations contain rich semantic knowledge that can further guide student learning. We therefore introduce a Teacher-Guided Feature Consistency Distillation (FCD) mechanism to enforce alignment between teacher and student feature spaces.

Let $F_{t}^{\left(i\right)}$ and $F_{s}^{\left(i\right)}$ denote the feature maps extracted from corresponding layers of the teacher and student networks for sample *i*. The feature consistency loss is defined as

$$L_{fcd}=\frac{1}{N}\sum_{i=1}^{N}{\left\|\phi \left(F_{t}^{\left(i\right)}\right)-\phi \left(F_{s}^{\left(i\right)}\right)\right\|}^{2}$$

where *ϕ*(⋅) denotes feature normalization and projection to eliminate scale discrepancies. To avoid excessive constraints when teacher predictions are uncertain, we introduce the following confidence-aware gating mechanism:

$$\omega_{l}=\sigma \left(\eta \left({\bar{c}}_{l}-\tau \right)\right)$$

where ${\bar{c}}_{l}$ is the average confidence at layer *l*, σ(⋅) is the sigmoid function, and *η* controls sensitivity. This adaptive weighting ensures that feature distillation is emphasized only when teacher outputs are reliable, thereby stabilizing student representation learning.

### 3.4. Deformable Pyramid Sparse Transformer (DPST)

**Figure 3:**
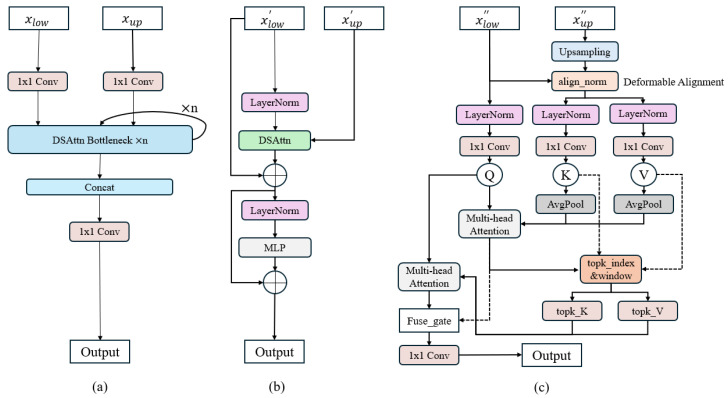
Overall architecture of the proposed Deformable Pyramid Sparse Transformer (DPST) module. (**a**) The framework of DPST. (**b**) Internal design of a DSAttn Bottleneck. (**c**) Detailed DSAttn process. In (**c**), align_norm normalizes concatenated multi-scale features and predicts spatial offsets for deformable alignment, ensuring precise correspondence between scales, topk_index identifies the most informative regions and their local windows for sparse fine-grained refinement, fuse_gate adaptively determines the fusion ratio of the two attention results at each spatial position through a 1 × 1 convolution and sigmoid gating.

Driver distraction detection requires sensitivity to subtle and localized visual patterns under complex lighting and occlusion conditions. To address the limitations of conventional convolutional pyramids, we propose a Deformable Pyramid Sparse Transformer (DPST) module, illustrated in Figure 3. DPST integrates deformable spatial alignment with staged sparse attention to enhance cross-scale semantic interaction.

**Figure 4:**
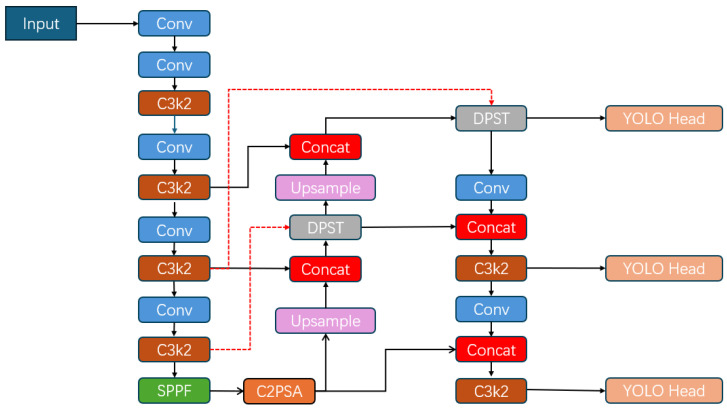
The final structure of our model after adding DPST.

Given low-level features $x_{low}\in R^{C_{1}\times H\times W}$ and high-level features $x_{up}\in R^{C_{2}\times \frac{H}{2}\times \frac{W}{2}}$, both are projected to a common embedding space via 1 × 1 convolutions. High-level features are then upsampled and aligned using deformable offsets Δ*p*:

$$x_{up}^{\prime \prime }\left(x\right)=\sum_{p\in \Omega }w_{p}\cdot x_{up}^{\prime }\left(x+∆p\right)$$

where Ω denotes the sampling window. After alignment, coarse global attention and fine sparse attention are applied sequentially, and the resulting features are fused through adaptive gating. The final output is obtained as

$$F_{out}={Conv}_{1\times 1}\left(\left[x_{low}^{\prime },y_{1},\ldots y_{n}\right]\right)$$

where *y*_*i*_ denotes outputs of attention blocks. DPST is integrated into both teacher and student networks, and the final architecture is shown in Figure 4. After deformable alignment, DPST performs staged sparse attention, consisting of a coarse global attention stage followed by a fine-grained local refinement stage. The resulting attention-enhanced features are concatenated and integrated via a 1 × 1 convolution. The detailed computational procedure of the proposed DPST module is summarized in Algorithm 1.
**Algorithm 1 Deformable Pyramid Sparse Transformer (DPST)**$\mathrm{Require}:\;\mathrm{Low}-\mathrm{level}\;\mathrm{feature}\;x_{low}\in R^{C_{1}\times H\times W}$$,\;\mathrm{high}-\mathrm{level}\;\mathrm{feature}\;x_{up}\in R^{C_{2}\times \frac{H}{2}\times \frac{W}{2}}$ $\mathrm{Ensure}:\;\mathrm{Enhanced}\;\mathrm{fused}\;\mathrm{feature}\;F_{out}\in R^{C_{2}\times H\times W}$ 1: **Step 1: Dual-Scale Embedding and Alignment** $2:\;f_{low}\leftarrow {\mathrm{Conv}}_{1\times 1}(x_{low})$ $3:\;f_{up}\leftarrow \mathrm{BilinearUpsample}({\mathrm{Conv}}_{1\times 1}(x_{up}),(H,W))$ $4:\;\Delta p\leftarrow {\mathrm{Conv}}_{3\times 3}([f_{low};f_{up}])$               ▷ Predcit deformable offsets $5:\;f_{align}\leftarrow \mathrm{GridSample}(f_{up},\mathrm{base}\_\mathrm{grid}+\Delta p)$          ▷ Deformable alignment 6: **Step 2: Hierarchical Sparse Attention** $7:\;Q\leftarrow {\mathrm{Conv}}_{1\times 1}^{Q}(f_{low})$ $8:\;[K,V]\leftarrow \mathrm{Split}({\mathrm{Conv}}_{1\times 1}^{KV}(f_{align}))$ $9:\;V\leftarrow V+{\mathrm{DepthwiseConv}}_{7\times 7}(f_{align})$ $10:\;K_{c},V_{c}\leftarrow {\mathrm{AvgPool}}_{2\times 2}(K,V)$ $11:\;A_{c}\leftarrow \mathrm{Softmax}(QK_{c}^{⊤}/\sqrt{d})$ $12:\;F_{c}\leftarrow A_{c}V_{c}$                ▷ Coarse cross-scale attention output 13: **Step 3: Local Refinement on Salient Regions** 14: if topk > 0 **then** $15:\;\;R\leftarrow \mathrm{TopK}\left(A_{c}\right)$             ▷ Select most informative regions $16:\;\;K_{r},V_{r}\leftarrow \mathrm{GatherLocal}\left(K,V,R\right)$ $17:\;\;A_{f}\leftarrow \mathrm{Softmax}(QK_{r}^{⊤}/\sqrt{d})$ $18:\;\;F_{f}\leftarrow A_{f}V_{r}$ $19:\;\;F\leftarrow \sigma ({\mathrm{Conv}}_{1d}([F_{c};F_{f}]))⊙F_{f}+(1-\sigma (\cdot ))⊙F_{c}$ 20: **else** $21:\;\;F\leftarrow F_{c}$ 22: **end if** 23: **Step 4: Iterative Refinement and Dense Fusion** $24:\;f_{0}\leftarrow f_{low}$ 25: for i =1 to n **do** $26:\;\;f_{i}\leftarrow f_{i-1}+{\mathrm{Conv}}_{1\times 1}(\mathrm{LayerNorm}(F))$ 27: **end for** $28:\;F_{cat}\leftarrow \mathrm{Concat}(f_{0},f_{1},\ldots ,f_{n})$ $29:\;F_{out}\leftarrow {\mathrm{Conv}}_{1\times 1}(F_{cat})$ $30:\;\mathrm{return}\;\;F_{out}$

### 3.5. Joint Loss Function and Optimization Strategy

The overall training objective combines supervised detection, pseudo-label supervision, and feature consistency distillation:

$$L_{total}=L_{sup}+\lambda_{1}L_{pseudo}+\lambda_{2}L_{fcd}$$

where *λ*_1_ and *λ*_2_ balance pseudo-label and distillation contributions. Training proceeds in stages: supervised learning dominates early training, pseudo-label supervision is gradually introduced via DS, and feature distillation is emphasized during later convergence. This progressive strategy ensures stable and effective knowledge transfer from labeled data to pseudo-supervised and feature-level learning.

## 4. Experimental Results and Discussion

### 4.1. Experiment Environment and Datasets

The data used in this study is the Distracted Driving Dataset from Roboflow [37]. The dataset comprises a total of 8864 annotated images spanning 12 distinct driver activity categories, including safe driving, talking on the phone, texting, drinking, reaching behind, talking to passengers, yawning, eyes closed, eyes open, nodding off, hair and makeup, and operating the radio. The dataset was originally split into 6860 samples for training, 1000 for validation, and 1004 for testing. In this experiment, we randomly extracted 20% of the labeled data from the original training set as labeled training data, and the remainder as unlabeled data, ensuring that data from each category was extracted.

All experiments were conducted on a cloud computing server equipped with an Intel Xeon Gold 6248R CPU, a NVIDIA A100-PCIE GPUs, and 144 GB RAM. The software environment was based on Ubuntu 20.04 LTS, and the deep learning framework was PyTorch 2.3.0. The input images were 640 × 640 pixels in size, and the SGD optimizer was used with an initial learning rate of 0.01 and a momentum of 0.937.

### 4.2. Evaluation Metrics

To comprehensively evaluate the effectiveness of the proposed semi-supervised driver distraction detection framework, we employ four widely adopted object detection metrics: Precision, Recall, mAP@0.5, and mAP@0.5:0.95. Precision measures the proportion of correctly predicted positive samples among all predicted positives, reflecting the model’s ability to suppress false alarms. Recall quantifies the proportion of ground-truth objects that are correctly detected, indicating the model’s sensitivity and missed-detection rate. The metric mAP@0.5 evaluates average precision under an Intersection over Union (IoU) threshold of 0.5, providing a general measure of detection performance, whereas mAP@0.5:0.95, averaged over IoU thresholds from 0.5 to 0.95 with a step size of 0.05, imposes stricter localization requirements and thus more rigorously reflects the detector’s robustness and boundary precision.

Precision and Recall jointly characterize the trade-off between detection accuracy and completeness, while the mAP metrics provide a holistic assessment of recognition and localization performance under varying matching strictness. In this study, mAP@0.5 and mAP@0.5:0.95 are treated as the primary evaluation criteria to analyze the contributions of individual modules and the overall effectiveness of the proposed framework, ensuring both practical relevance and academic rigor.

### 4.3. Comparison with State-of-the-Art Methods

Table 1 presents a quantitative comparison between the proposed semi-supervised framework and several representative fully supervised driver distraction detection methods, including RT-DETR [38], Tian et al. [39], YOLO13 [40], and the baseline YOLOv11.

As shown in Table 1, the proposed method achieves the best overall detection performance in terms of mAP@0.5 (66.66%) and mAP@0.5:0.95 (48.59%), indicating improved robustness under both relaxed and strict IoU criteria. Compared with the fully supervised YOLOv11 baseline, the proposed framework improves mAP@0.5 by 0.57 percentage points and mAP@0.5:0.95 by 0.56 percentage points, demonstrating that the semi-supervised learning strategy effectively enhances both coarse detection capability and fine-grained localization accuracy.

Some fully supervised methods, such as RT-DETR and Tian et al., achieve relatively high Precision values (78.19% and 78.23%, respectively), which suggests conservative prediction behavior with strong confidence filtering. However, this comes at the cost of substantially lower Recall, indicating a large number of missed detections. In safety-critical driver distraction detection scenarios, such recall degradation is undesirable, as undetected risky behaviors may directly affect system reliability.

The YOLOv11 baseline exhibits the highest Recall (62.95%), reflecting strong coverage of driver behavior regions, but its Precision remains limited (58.00%), indicating susceptibility to background interference and ambiguous in-cabin cues. In contrast, the proposed semi-supervised framework increases Precision to 61.43%, achieving a 3.43% absolute gain over YOLOv11, while maintaining a comparable Recall level. This balanced improvement suggests that the proposed method effectively suppresses false positives without significantly increasing missed detections.

Overall, these results indicate that the proposed semi-supervised approach alleviates the precision–recall imbalance commonly observed in fully supervised detectors and provides more reliable performance for fine-grained, real-world driver distraction detection with only marginal increases in model complexity.

### 4.4. Effectiveness of the DPST Module

To evaluate the contribution of the proposed Deformable Pyramid Sparse Transformer (DPST), we conduct controlled experiments comparing YOLOv11 with and without the DPST module. In addition to the baseline YOLOv11, we further include PST (Pyramid Sparse Transformer) [42] as a structural comparison, which employs sparse attention over fixed pyramid grids without deformable alignment or adaptive gating. The quantitative results are summarized in Table 2.

As shown in Table 2, introducing PST does not lead to consistent performance improvement over the baseline, indicating that sparse attention alone is insufficient for robust feature modeling in driver distraction scenarios characterized by spatial and scale variations. In contrast, DPST achieves a notable precision improvement, increasing Precision from 58.00% to 61.49%, which demonstrates its enhanced ability to suppress false positives. This gain can be attributed to the deformable alignment mechanism and adaptive gating, which enable more accurate cross-scale feature correspondence and emphasize semantically meaningful regions.

Moreover, mAP@0.5:0.95 improves from 48.03% to 48.19%, suggesting that DPST provides more accurate localization under stricter IoU thresholds. Although Recall decreases from 62.95% to 59.23%, this behavior reflects the design characteristic of DPST, which prioritizes high-confidence and well-aligned features while suppressing ambiguous or noisy responses. Such a precision-oriented behavior is desirable in safety-critical applications and also motivates the integration of DPST with complementary mechanisms, such as semi-supervised learning, to recover recall while preserving the precision gains.

### 4.5. Ablation Study of Proposed Components

To further analyze the individual contributions of each proposed component, we conduct a comprehensive ablation study, the results of which are reported in Table 3.

Starting from the baseline YOLOv11, the introduction of DPST yields improvements in both mAP@0.5 and mAP@0.5:0.95, confirming the effectiveness of deformable multi-scale feature alignment and sparse attention in enhancing feature representation. Precision also improves modestly, demonstrating DPST’s ability to suppress false positives caused by blurred boundaries or background clutter.

The addition of the Pseudo-Label Optimization Strategy (PLOS) results in a substantial Precision increase from 50.69% to 59.85%, alongside notable gains in mAP@0.5:0.95. This highlights the critical role of high-quality pseudo-label generation, scheduling, and confidence-aware weighting in stabilizing semi-supervised training and preventing noise accumulation.

Finally, incorporating Feature Consistency Distillation (FCD) leads to the best overall performance. The final model achieves 66.66% mAP@0.5 and 48.59% mAP@0.5:0.95, validating that feature-level semantic alignment between teacher and student networks effectively mitigates pseudo-label ambiguity and improves representation stability. FCD acts as an implicit regularizer, ensuring coherent feature distributions and enhancing generalization in complex driving environments.

### 4.6. Qualitative Analysis and Visualization

**Figure 5:**
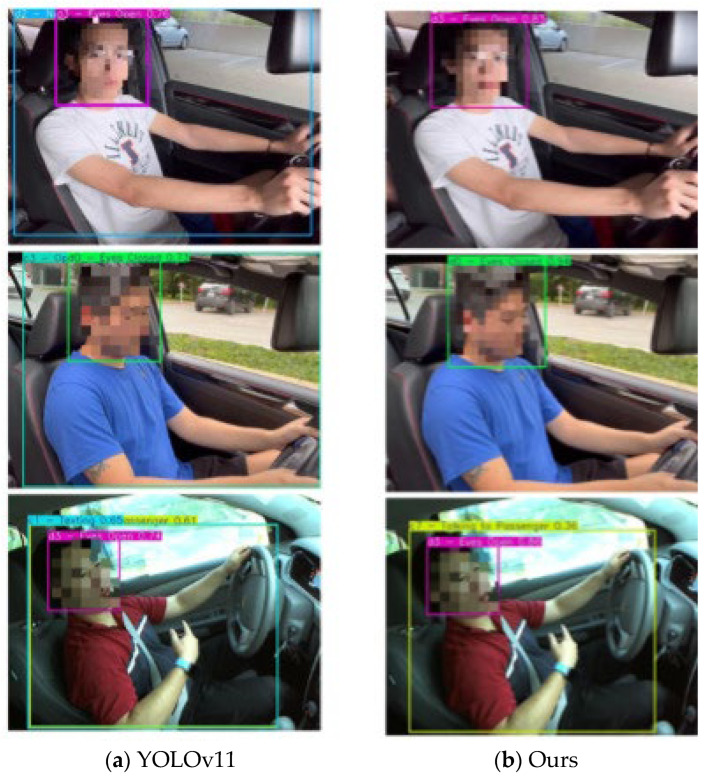
Qualitative comparison of YOLOv11 and the proposed method in the driver distraction detection task. (**a**) Detection results of the original YOLOv11; (**b**) Output of the proposed semi-supervised detection model.

To further illustrate the quantitative improvements reported in Table 1, Table 2 and Table 3, Figure 5 presents qualitative comparisons between YOLOv11 and the proposed semi-supervised framework under representative driving scenarios. Figure 5 presents a qualitative comparison between YOLOv11 and the proposed semi-supervised model on representative driving scenarios. As shown in Figure 5a, YOLOv11 frequently produces low-confidence predictions and incorrect classifications, such as confusing Eyes Open, Eyes Closed, and Nodding Off, or misclassifying ambiguous hand gestures as Texting. These errors highlight the difficulty of distinguishing subtle driver behaviors using purely supervised learning.

In contrast, the proposed method (Figure 5b) consistently generates accurate and high-confidence predictions across the same scenarios, demonstrating improved robustness to ambiguous postures and overlapping visual cues. Specifically, the reduction in incorrect classifications and low-confidence predictions in Figure 5b corresponds to the observed Precision improvement, while the tighter and more accurate bounding boxes are consistent with the gain in mAP@0.5:0.95.

**Figure 6:**
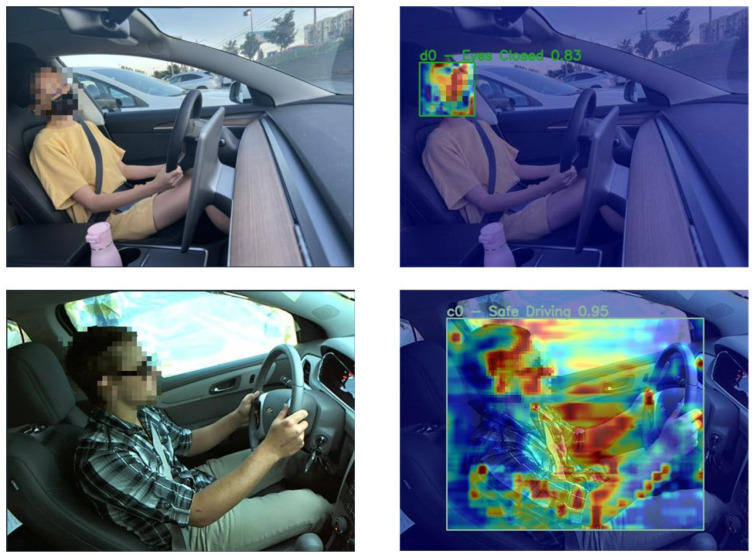
Driver distraction detection heat map results and visualization.

Figure 6 further visualizes the model’s attention behavior using Grad-CAM heatmaps. In the Eyes Closed scenario, the model accurately focuses on the eye region with a confidence score of 0.83, while in the Safe Driving scenario, attention is concentrated on the face and hands with a confidence of 0.95. These visualizations confirm that the proposed framework not only improves detection accuracy but also exhibits strong interpretability by attending to semantically meaningful regions relevant to driver state assessment. This focused attention behavior explains the improved localization accuracy and reduced false detections reflected in the mAP@0.5:0.95 and Precision metrics.

### 4.7. Practical Applicability Under Driver Behavior Variability

In practical driver monitoring systems, significant differences in driver behavior pose a major challenge to reliable distraction detection. These differences stem from variations in driver appearance, posture, driving habits, interaction patterns, and environmental conditions, leading to significant intra-class variability even within the same distraction category. In real-world scenarios, this variation typically manifests as increased false positive rates and localization instability, especially for fine-grained behaviors with subtle and localized visual cues.

Quantitatively, the impact of driver behavior differences is reflected in the trade-off between precision and recall, and performance degradation under stricter localization criteria. As shown in Table 1, fully supervised detectors exhibit drastically different predictive behaviors. Methods such as those proposed by RT-DETR and Tian et al. achieve high precision but significantly low recall, indicating a conservative prediction strategy that misses a large number of valid driving behaviors. In contrast, recall-oriented detectors tend to introduce more false positives when faced with diverse driving patterns and ambiguous postures. The proposed semi-supervised framework demonstrates greater robustness in dealing with such variations. Compared to the fully supervised YOLOv11 baseline method, our method improves precision from 58.00% to 61.43%, achieving an absolute improvement of 3.43% while maintaining a similar recall. This demonstrates that the proposed method can more effectively suppress false positives caused by atypical driver behavior and ambiguous visual cues. Furthermore, mAP@0.5:0.95 improves from 48.03% to 48.59%, reflecting enhanced localization stability under stricter IoU constraints, which is particularly important when the scale and spatial configuration of interfering cues vary among different drivers.

From a representational perspective, the Deformable Pyramid Sparse Transformer (DPST) further enhances this robustness by achieving spatial adaptation and fine-grained feature modeling. By establishing a deformable cross-scale alignment and sparse attention mechanism, DPST enables the network to focus on semantically relevant regions, such as hands, head, and eyes, even with pose and viewpoint variations. The quantitative improvements in mAP@0.5:0.95 and precision are consistent with the qualitative results shown in Figure 5 and Figure 6, indicating that the proposed method reduces false detections and provides more accurate bounding boxes for subtle perturbations. Furthermore, the Feature Consistency Distillation (FCD) mechanism enhances robustness by stabilizing the semantic representations between the teacher and student models. As shown in the ablation experiments (Table 3), the introduction of FCD significantly improves mAP@0.5:0.95, demonstrating that feature-level consistency helps mitigate the impact of noisy false labels often caused by rare or unconventional behavioral patterns.

From a deployment perspective, practical applications also require performance improvements without significantly increasing model complexity. As shown in Table 1, compared to the YOLOv11 baseline model, the proposed framework only slightly increases computational costs, with the number of parameters increasing from 2.6 million to 2.7 million and GFLOPS increasing from 6.3 to 6.6. This slight overhead indicates that improving the model’s robustness to changes in driver behavior is not simply achieved by expanding model capacity but through more effective learning strategies and feature modeling mechanisms. This moderate increase in complexity aligns with the constraints of in-vehicle edge devices, supporting the feasibility of deploying the proposed method in a real-world driver monitoring system.

## 5. Conclusions

This paper presents a novel semi-supervised driver distraction detection framework that effectively exploits large-scale unlabeled driving behavior data to enhance the accuracy, robustness, and generalization capability of distracted driving recognition systems. Built upon a lightweight YOLOv11 detector, the proposed framework is specifically designed to address the challenges of fine-grained driver behavior analysis under limited annotation conditions.

To improve sensitivity to subtle and localized driver actions, we introduce a Deformable Pyramid Sparse Transformer (DPST) module that combines deformable geometric alignment with staged sparse attention. This design enables the model to capture fine-grained local cues—such as eye closure, hand gestures, and head posture—while preserving global contextual information across multiple feature scales. In addition, a set of adaptive pseudo-label optimization strategies, including Category-Aware Pseudo-Labeling (CAPL), Delayed Scheduling (DS), and Confidence-Weighted Pseudo-Label Loss (CWPL), is incorporated to dynamically regulate pseudo-label selection, temporal introduction, and loss contribution. These mechanisms effectively suppress noise accumulation and stabilize semi-supervised training. Furthermore, the proposed Feature Consistency Distillation (FCD) module enforces semantic alignment between teacher and student feature representations, facilitating reliable knowledge transfer and improving representation stability in the presence of imperfect pseudo-labels.

Extensive experiments conducted on the Roboflow Distracted Driving Dataset demonstrate the effectiveness of the proposed approach. Compared with a fully supervised baseline, the proposed framework consistently achieves higher mAP@0.5 and mAP@0.5:0.95, validating its superior capability in both coarse detection and fine-grained localization of distracted driving behaviors. The results confirm that the integration of adaptive semi-supervised learning and deformable multi-scale feature modeling provides a practical and effective solution for real-world driver monitoring systems.

In future work, we plan to extend this framework toward multimodal driver monitoring by incorporating complementary cues from infrared imagery and physiological signals to further enhance robustness under challenging illumination and occlusion conditions. Additionally, we aim to explore the integration of contrastive self-refinement and uncertainty-aware pseudo-labeling to improve adaptability and reliability in dynamic, real-world intelligent vehicle environments.

## Figures and Tables

**Table 1:** Comparison of overall performance of different methods.

Method	Precision (%)	Recall (%)	mAP@0.5	mAP@0.5:0.95	Parameters	GFLOPs
Supervised algorithms						
RT-DETR [38]	78.19	54.72	60.79	43.97	20	60
Tian’s [39]	78.23	51.59	62.01	47.24	2.5	5.8
RT-DETRv2 [41]	66.37	64.57	60.24	44.91	20	60
YOLO13 [40]	49.58	58.67	60.83	46.47	2.5	6.4
YOLOv11	58.00	62.95	66.09	48.03	2.6	6.3
Semi-supervised algorithms						
**Ours**	61.43	58.96	66.66	48.59	2.7	6.6

**Table 2:** Performance evaluation of the DPST module integrated into YOLOv11.

Method	Precision (%)	Recall (%)	mAP@0.5	mAP@0.5:0.95
YOLOv11	58.00	62.95	66.09	48.03
YOLOv11 + PST	54.19	64.21	63.23	47.86
YOLOv11 + DPST	61.49	59.23	63.44	48.19

**Table 3:** Ablation study of the proposed modules.

Method	Precision (%)	Recall (%)	mAP@0.5	mAP@0.5:0.95
YOLOv11	50.12	59.10	61.56	43.59
YOLOv11 + DPST	50.69	58.60	62.29	44.58
YOLOv11 + DPST + PLOS	59.85	59.74	62.30	46.08
YOLOv11 + DPST + PLOS + FCD	61.43	58.96	66.66	48.59

## Data Availability

All datasets used in this study were sourced from publicly available on Roboflow at https://universe.roboflow.com/ipylot-project/distracted-driving-v2wk5 (accessd on 10 November 2025).
